# Association between the metabolic syndrome and its components and gait speed among U.S. adults aged 50 years and older: a cross-sectional analysis

**DOI:** 10.1186/1471-2458-6-282

**Published:** 2006-11-14

**Authors:** Catherine A Okoro, Yuna Zhong, Earl S Ford, Lina S Balluz, Tara W Strine, Ali H Mokdad

**Affiliations:** 1 National Center for Chronic Disease Prevention and Health Promotion, Centers for Disease Control and Prevention, Atlanta, Georgia, USA

## Abstract

**Background:**

To examine the relationship between the metabolic syndrome and its components and gait speed among older U.S. men and women. Whether these associations are independent of physical activity was also explored.

**Methods:**

Eight hundred and thirty-five men and 850 women aged ≥50 years from the continuous National Health and Nutrition Examination Survey 1999–2002 were examined. We used the definition of the metabolic syndrome developed by the U.S. National Cholesterol Education Program Adult Treatment Panel III. Gait speed was measured with a 6.10-meter timed walk examination.

**Results:**

The prevalence of the metabolic syndrome was 40.2% in men and 45.6% in women (*P *= .127). The prevalence of gait speed impairment was 29.3% in men and 12.5% in women (*P *< .001). No association was found between the metabolic syndrome and gait speed impairment. After including the individual components of the metabolic syndrome in a logistic model adjusted for age and leisure-time physical activity, abdominal obesity, low HDL cholesterol, and high fasting glucose were significantly associated with gait speed impairment among women (adjusted odds ratio [AOR] = 0.48, 95% confidence interval [CI] = 0.26 to 0.89; AOR = 2.26, 95% CI = 1.08 to 4.75; and AOR = 2.05, 95% CI = 1.12 to 3.74, respectively). Further adjustment for race/ethnicity, education, smoking status, alcohol consumption, arthritis status, and use of an assistive device attenuated these associations; among women, abdominal obesity and low HDL cholesterol remained significantly associated with gait speed impairment (AOR = 0.37, 95% CI = 0.18 to 0.76 and AOR = 2.45, 95% CI = 1.07 to 5.63, respectively) while the association between hyperglycemia and impaired gait speed attenuated to nonsignificance.

**Conclusion:**

Among women, gait speed impairment is associated with low HDL cholesterol and inversely with abdominal obesity. These associations may be sex-dependent and warrant further research.

## Background

Mobility is critical to maintaining independence and social interaction in the later years of life and is an essential aspect of quality of life [1]. Persons with poor lower-extremity function, slow gait speed, and particularly those with walking disability, are at higher risk for chronic disease, hospitalization, and death than their peers who walk normally [1-3]. Several general impairments and acute and chronic conditions have been linked to poor lower-extremity function and are often multifactorial [1,4,5]. One such condition, hyperglycemia, has been found to be a strong predictor of disability and poor physical performance among older U.S. adults [6,7]. Other conditions, such as heart disease, stroke, and hypertension, have also been reported to predispose older adults to functional decline [8,9]. Furthermore, modifiable health behaviors, such as level of physical activity, may play an important role in the prevention of chronic conditions that could lead to functional limitation and disability [10,11]. Identifying risk factors for slow gait speed and developing appropriate interventions may be the best approach for preventing decrements in gait speed and delaying its progression.

One such risk factor may be the metabolic syndrome, which the U.S. National Cholesterol Education Program Adult Treatment Panel III (NCEP/ATP III) defines as the presence of three or more of the following conditions: abdominal adiposity, high blood pressure, hypertriglyceridemia, low levels of high-density lipoprotein (HDL) cholesterol, or high fasting glucose [12]. Approximately 27% of all U.S. adults are estimated to have the metabolic syndrome, but the prevalence is even higher among older men and women: 33.0% and 30.6%, respectively, among those aged 40 to 59 years and 39.7% and 46.1%, respectively, among those aged 60 years or older [13]. With the epidemics of obesity and diabetes in the United States, the metabolic syndrome is expected to be even more prevalent in the future [13,14]. Persons with the metabolic syndrome have been shown to be at increased risk for cardiovascular disease and diabetes mellitus, as well as for death from cardiovascular disease and from all causes [15,16]. However, there is minimal research on the association between the metabolic syndrome and its individual components and gait speed.

The purpose of this study was to assess whether the metabolic syndrome and its individual components are associated with gait speed in a national sample of U.S. men and women aged 50 years and older. Whether these associations are independent of physical activity was also examined.

## Methods

### Design and data collection

Beginning in 1999, the Centers for Disease Control and Prevention's (CDC's) National Health and Nutrition Examination Survey (NHANES) became a continuous survey of the U.S. civilian noninstitutionalized population based on a complex, multistage sampling design. This report is based on results of the first four years of the continuous survey, 1999–2000 and 2001–2002. Informed consent was obtained from all participants, and the CDC Institutional Review Board approved the protocol. A detailed description of the continuous NHANES is reported elsewhere [17].

### Measures

The survey included an interview conducted in participants' households and an examination at a mobile examination center (MEC). Information obtained during the interview included demographic data such as sex, age, race and ethnicity, years of education completed, and household income. Interviewers also asked survey participants about their health conditions and health-related behaviors, including questions about smoking; physical activity; hypertension; and use of antihypertensive medication, diabetes medication, and insulin. After the household interview, participants were asked to undergo a health examination at a MEC.

In accordance with the NCEP/ATP III criteria [12], we considered participants to have the metabolic syndrome if they met three or more of the following criteria: 1) abdominal obesity: waist circumference >102 cm in men and >88 cm in women; 2) hypertriglyceridemia: ≥150 mg/dL (1.695 mmol/L); 3) low levels of HDL cholesterol: <40 mg/dL (1.036 mmol/L) in men and <50 mg/dL (1.295 mmol/L) in women; 4) systolic blood pressure/diastolic blood pressure: ≥130/85 mm Hg; and 5) high fasting glucose: ≥110 mg/dL (≥6.10 mmol/L). Participants who reported current use of antihypertensive or antidiabetic medication (insulin or oral agents) were counted as having high blood pressure or diabetes, respectively.

Waist circumference was measured according to standard protocol by a trained NHANES 1999–2002 staff member to the nearest millimeter [18]. During the health examination, up to four blood pressure readings were obtained. If three or more blood pressure readings were obtained, we excluded the first reading and used the average of the remaining readings; if two readings were obtained, we used the last reading; and if only one reading was obtained, we used that. Participants' body mass index (BMI) was calculated from their measured weight and height (weight [kg]/height [m^2^]). Serum triglycerides (after hydrolysis to glycerol) and HDL cholesterol (after precipitation with a heparin sulfate and MnCl_2 _solution) were measured enzymatically. Plasma glucose concentrations were measured by using an enzymatic reaction.

Among participants aged 50 years and older, gait speed was measured by observing them take a 20-foot (6.10 meter [m]) walk at their usual pace and timing them with a hand-held stopwatch to the nearest hundredth of a second. The stopwatch was started when a participant's foot first touched the floor across the start line and stopped when the participant's foot first touched the floor across the finish line. Participants were permitted to use a walking device such as a walker or cane. We converted the results into speed (meters per second [m/s]) to facilitate comparison to other studies. Bohannon et al.'s [19] reference values for comfortable gait speed among older adults were used to define normal gait speed; men with a gait speed <0.943 m/s and women with a gait speed <0.713 m/s were classified as having a gait speed impairment.

Covariates included age (continuous), sex, race or ethnicity (non-Hispanic white, non-Hispanic black, Mexican American, other), education (<high school, ≥high school), metabolic equivalent (MET) units of leisure-time physical activity (0, >0-<22.5, 22.5-<35, ≥35; weekly), smoking status (current, former, never), alcohol consumption (none, moderate, heavy), arthritis status (yes, no), and use of an assistive device (yes, no). Participants were asked to report the frequency and the duration that they spent participating in 48 leisure-time physical activities of moderate or vigorous intensity the month before the survey [20-23]. Each physical activity was assigned an intensity value by NHANES 1999–2002 that represents the ratio of the energy expenditure of the activity to a standard resting metabolic rate of 1.0 [21,23,24]. We multiplied each activity session by its corresponding intensity value and summed this product for all activity types for each participant: ∑_i_(number of times * METs)_i_, where i = a specific leisure-time physical activity from 1 to 48. This produced an intensity-weighted frequency value for the past month that we then converted into an average weekly value. We then created 4 categories, based on recommended levels of physical activity [25], that corresponded to 1) no leisure-time physical activity (0 METs/week); 2) insufficient leisure-time physical activity (>0-<22.5 METs/week, where 22.5 METs/week represents the minimum recommended level based on the MET value of 5 days of brisk walking (4.5 METs)) [11,24,26]; 3) meeting the minimum recommendation (22.5-<35 METs/week); and 4) highly active (≥35 METs/week; where 35 METs/week represents 5 days of vigorous aerobic activity). A current smoker was defined as someone who reported smoking at least 100 cigarettes in their lifetime and currently smokes cigarettes. A former smoker was defined as someone smoking at least 100 cigarettes in their lifetime who reported that they no longer smoked cigarettes. Those who had not smoked 100 cigarettes in their lifetime were defined as never having smoked. Alcohol consumption was based on the National Institute on Alcohol Abuse and Alcoholism's guide for screening for heavy drinking among older adults [27]. A non-drinker was defined as someone who reported not drinking any alcoholic beverages in the past 12 months. Men aged 50–65 years were considered moderate drinkers if they consumed ≤2 drinks per day on average, as were men aged 66 years or older and women aged 50 years or older who consumed ≤1 drink per day. Men aged 50–65 years were considered heavy drinkers if they consumed more than 2 drinks per day on average, as were men aged 66 years or older and women aged 50 years or older who consumed more than 1 drink per day. A drink was considered as a 12-oz beer, a 4-oz glass of wine, or an ounce of liquor. In NHANES 1999–2002 no distinction was made between different types of alcoholic drinks. Participants were considered to have arthritis if they answered "yes" to the question, "Has a doctor or other health professional ever told you that you had arthritis?" Subjects who were observed using a cane, walker, or another walking aid during the 20-foot (6.10 m) timed walk test were categorized as using an assistive device.

### Statistical analysis

Among both men and women, we calculated the mean values of continuous variables and percentages of categorical variables of participant's general and clinical characteristics, and across those with different number of metabolic syndrome components; we calculated the age-adjusted prevalence of gait speed impairment across participants with different number of metabolic syndrome components; and we calculated the prevalence of each component of the metabolic syndrome and for the syndrome as a whole by gait speed impairment status. To age-adjust statistics, we directly adjusted to the U.S. population ≥50 years old in the year 2000. We determined whether differences in these characteristics were statistically significant by using a Chi-squared test for categorical variables and a *t *test for continuous variables. The unadjusted odds ratios (ORs) were calculated for the association between gait speed impairment and the metabolic syndrome and each of its individual components using logistic regression. We then estimated the OR between gait speed impairment and each of the 5 components of the metabolic syndrome (abdominal obesity, hypertriglyceridemia, low HDL cholesterol, hypertension, and high fasting glucose, respectively) after adjustment for age and METs/week of leisure-time physical activity (Model 1). Model 1 was further adjusted to include all metabolic syndrome components (Model 2). Finally, in the full model, we include race/ethnicity, education, and all variables that were moderately associated with the outcome variable during univariate analyses. The full model was then reduced by the stepwise elimination of variables with *P *values ≥ .05 with the exception of important confounders (Model 3). We assessed the fit of the final model using the Hosmer-Lemeshow goodness-of-fit test [28]. SUDAAN 9.0.1 (Research Triangle Institute, 2005) was used to account for the survey's complex sampling design. All statistical inferences were based on a significance level of *P *< .05. Prevalence estimates were calculated by using the sampling weights so that the estimates were representative of the civilian, noninstitutionalized U.S. population.

Because concentrations of plasma glucose and serum triglycerides were measured using reference analytic methods only for participants who attended the morning examination, we limited the analyses to 1,915 subjects aged ≥50 years who attended the morning examination and who had fasted ≥8 hours. Participants were excluded who did not have complete measurements for all components of the metabolic syndrome (N = 131) or had missing data on the 20-foot (6.10 m) timed walk test (N = 99). Data from the remaining 1,685 participants (835 men and 850 women) were included in the analyses. The age and sex distributions of those included in the analyses were not significantly different from those who were excluded (both *P *> .05) [see Additional file 1].

## Results

Table 1 shows general and clinical characteristics of participants by gender. Men and women had similar percentages of participants who were white, black, Mexican American, or another race; had graduated from high school; were obese; or used an assistive device. Men were younger and were more likely to be current or former smokers and to be heavy drinkers. Women were more likely to not participate in physical activity during leisure time, to never have smoked, to be non-drinkers, and to have arthritis than men. Mean waist circumference and triglycerides were outside the normal range in both men and women, as was mean systolic blood pressure in women and mean fasting glucose in men. Men had higher values of waist circumference and fasting glucose and lower levels of systolic blood pressure and total and HDL cholesterol. Mean HDL cholesterol was within the normal range in both men and women.

Overall, 40.2% men and 45.6% women (*P *= 0.127) were defined as having the metabolic syndrome. Among men, 11.0% had none of the components of the metabolic syndrome, 19.7% had 1, 29.1% had 2, 22.8% had 3, 12.0% had 4, and 5.4% had all the components. For women, the figures were 8.4%, 21.8%, 24.3%, 21.6%, 17.6%, and 6.4%, respectively. As anticipated, BMI, waist circumference, systolic blood pressure, low HDL cholesterol, fasting glucose, and triglycerides significantly increased with increasing number of metabolic syndrome components in both men and women. In participants with the metabolic syndrome, high fasting glucose was more frequent in men (*P *< .001) and abdominal obesity and high blood pressure were more frequent in women (both *P *< .05) (data not shown).

**Figure 1:**
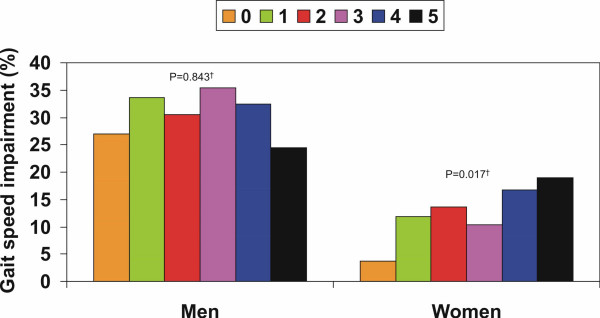
Age-adjusted prevalence of gait speed impairment^a ^according to number of metabolic syndrome components, NHANES 1999–2002. NHANES, National Health and Nutrition Examination Survey. ^a^Age-adjusted to the 2000 U.S. standard population. ^†^Denotes test for trend (*P*-value).

Table 2 provides the prevalence of individual components of the metabolic syndrome by gender and gait speed impairment status. Overall, the prevalence of gait speed impairment was 29.3% in men and 12.5% in women (*P *< .001). Both men and women with impaired gait speed had a higher prevalence of hypertension than those with normal gait speed; however, there was no statistical difference in the age-adjusted prevalence of this component of the metabolic syndrome. Women with impaired gait speed had a higher age-adjusted prevalence of low HDL cholesterol, elevated fasting glucose, and the metabolic syndrome itself than those with normal gait speed. In addition, the age-adjusted prevalence of gait speed impairment significantly increased with increasing number of components of the metabolic syndrome among women (Figure 1).

In both unadjusted and adjusted analyses, no association was found between the metabolic syndrome (i.e., the presence of at least 3 of the 5 components of the syndrome) and gait speed impairment (Table 3). When including the various components of the metabolic syndrome separately in the unadjusted model, both men and women with hypertension were more likely to have gait speed impairment than those without hypertension; and women with high fasting glucose were 2.42 times more likely to have impaired gait speed than those without high fasting glucose. Adjustment for age and level of physical activity attenuated the association between high blood pressure and gait speed impairment to nonsignificance in both men and women; however, among women the association between high fasting glucose and gait speed impairment remained significant (adjusted OR [AOR] = 1.90, 95% confidence interval (CI) = 1.14 to 3.17) and low HDL cholesterol achieved significance (AOR = 1.88, 95% CI = 1.04 to 3.41) (Table 3, model 1). After further adjustment for the other metabolic components, hyperglycemia and low HDL cholesterol continued to be significantly associated with gait speed impairment among women; and those who had abdominal obesity were less likely to have gait speed impairment than those who did not have abdominal obesity (AOR 0.48; 95% CI = 0.26 to 0.89) (Table 3, model 2). The associations between abdominal obesity and low HDL cholesterol and gait speed impairment among women continued to be significant after adding race/ethnicity, education, smoking status, alcohol consumption, arthritis status, and use of an assistive device to the adjusted 2 model (abdominal obesity: AOR = 0.37, 95% CI = 0.18 to 0.76; low HDL cholesterol: AOR = 2.45, 95% CI = 1.07 to 5.63) (Table 3, model 3). In the fully adjusted model, the association between hyperglycemia and gait speed impairment attenuated and was no longer statistically significant among women (AOR = 1.84, 95% CI = 0.84 to 4.03).

## Discussion

In this cross-sectional survey of the U.S. general population aged 50 years or older, we found no association between the metabolic syndrome and impaired gait speed among either gender. However, our findings suggest that among women, gait speed impairment is associated with abdominal obesity and low HDL cholesterol even after adjusting for age, level of physical activity, other components of the metabolic syndrome (i.e., hypertriglyceridemia, high blood pressure, and high fasting glucose), and other confounders. Specifically, women with abdominal obesity are significantly less likely to have gait speed impairment than those without abdominal obesity, and women with low HDL cholesterol are significantly more likely than those without this disorder to have gait speed impairment.

HDL cholesterol may have further clinical relevance besides the inverse relationship with coronary heart disease [12]. In fact, the results of this study are consistent with findings of previous studies that have linked low values of HDL cholesterol to chronic illness, severe clinical conditions, and functional disability in the elderly [29-31]. For example, Zuliani et al.[29] found that, in a longitudinal study of institutionalized adults over the age of 65 years, low HDL cholesterol levels were strongly associated with severe disability. In addition, their longitudinal data suggests that low HDL cholesterol is a potential indicator for ongoing disability in basic activities of daily living [29]. Our finding that gait speed impairment is associated with low HDL cholesterol after adjustment for level of physical activity may be explained by previous studies that reported physical activity to have little effect on increases in HDL cholesterol in the absence of weight loss [32,33].

The results of this study are not consistent with findings of previous studies concerning the relationship between abdominal obesity and impaired gait speed [34,35]. For example, Angleman et al. [34] reported that, in periretirement age women (i.e., aged 55 to 74 years), increases in waist circumference best predicted risk for slow gait speed and other disability outcomes. One possible explanation for our inconsistent study results may be the applied cutpoint used to define impaired gait speed; Angleman et al. defined slow gait speed as <0.6 m/s while we used <0.713 m/s. The inter-relationships between body size and predictors of morbidity and mortality in older adults (that is, ≥65 years) are controversial; and the confounding of weight loss with chronic disease and age should be considered when evaluating these associations [36,37]. More research is needed into the association between abdominal obesity and gait speed as well as the most appropriate anthropometric measure to use in examining the relationship.

Previous studies have reported an association between hyperglycemia and impaired gait speed [1,4-7]. Many older adults with diabetes do not attain glycemic control targets [6], and effective blood-glucose control has also been shown to delay the onset or slow the progression of diabetic retinopathy, nephropathy, and neuropathy in adults with insulin-dependent type 2 diabetes [38]. Our findings demonstrate that gait speed impairment is not significantly associated with high fasting glucose in men. On the other hand, a significant association persisted after adjustment for age, level of physical activity, and the other components of the metabolic syndrome among women. Even so, further adjustment for race/ethnicity, education, smoking status, alcohol consumption, arthritis, and use of an assistive device attenuated the association to nonsignificance.

Controlling lifestyle-related behaviors such as amount of physical activity, consuming a balanced diet, and maintaining a healthy weight, may prevent, delay, or improve components of the metabolic syndrome as well as mobility loss [1,7,11,12,33,39-41]. Indeed, the role of physical activity and weight loss (when appropriate) cannot be underemphasized for their capacity to improve health risks [32]; increased levels of physical activity are inversely related to morbidity and mortality independent of metabolic disorders and obesity [33]. In addition, comorbidities such as diabetes and coronary heart disease have also been associated with strength impairment [42,43], though strength impairment and other impairments may also be prevented through strategies that incorporate physical activity, self-care, and good chronic disease management [43]. Furthermore, earlier studies have demonstrated a relationship between lower extremity muscle strength and gait speed [19,44,45]; adults in their eighties and nineties have achieved increased gait speed through muscle strength training [44].

This study has several limitations. First, since components of the metabolic syndrome are correlated, we are estimating controlled direct effects under the assumption of a properly specified model and no unmeasured confounding [46]. For example, we may not have totally removed the confounding effects of physical activity, which has a strong relationship to the individual components of the metabolic syndrome and gait speed. Second, we were unable to determine whether participants' impaired gait speed was progressive or catastrophic, and the association between the metabolic syndrome and its components and gait speed may differ depending on the cause of the impairment. Third, lower-extremity-strength and balance, both prerequisites for the ability to walk [43], were not addressed in our analyses; nor was weight loss which may modify the effect of level of physical activity on our results [32,33]. Fourth, because continuous NHANES 1999–2002 was conducted among the noninstitutionalized U.S. population, our results probably underestimate the prevalence of gait speed impairment and the metabolic syndrome and its components among the total U.S. population. Finally, because of the cross-sectional study design, we could not determine whether the relationship between the metabolic syndrome and its individual components and gait speed impairment was a causal one.

## Conclusion

In summary, we found no association between the metabolic syndrome and gait speed impairment. However, among women, we did find a significant association between low HDL cholesterol and gait speed impairment, and an inverse association with abdominal obesity. These associations may be sex-dependent and warrant further research. If these conditions are not properly addressed at the population level, their increasing prevalence may translate into increased morbidity and mortality and threaten both independent living and quality and years of healthy life [1-4,13,14,19,43].

## Competing interests

The author(s) declare that they have no competing interests.

## Authors' contributions

CO and EF conceived of the study. YZ, EF, LB, TS, and AM provided feedback and suggestions for the analyses and YZ completed the analyses. CO synthesized analyses and led the writing. All authors helped to conceptualize ideas, interpret findings, reviewed drafts of the manuscript, and read and approved the final manuscript.

## Pre-publication history

The pre-publication history for this paper can be accessed here:



## Supplementary Material

Additional File 1 Sex and age distribution of study participants and excluded participants, NHANES 1999–2002. Sex and age characteristics of participants included and excluded from the study. Click here for file

## Figures and Tables

**Table 1:** General and clinical characteristics of U.S. adults aged ≥50 by gender, NHANES 1999–2002

Characteristic	Men (N = 835) % (or mean) ± SE	Women (N = 850) % (or mean) ± SE	P
Age, years, mean	62.8 ± 0.4	64.1 ± 0.3	0.0083
Non-Hispanic white	83.5 ± 1.9	81.5 ± 2.6	0.1703
Non-Hispanic black	6.3 ± 1.0	6.8 ± 1.3	0.5443
Mexican American	3.7 ± 0.7	3.4 ± 0.8	0.4768
Other (other & other Hispanic)	6.5 ± 1.4	8.3 ± 2.4	0.2099
≥High school education	76.9 ± 1.8	75.0 ± 1.5	0.2955
Gait speed impairment	29.3 ± 2.6	12.5 ± 1.3	0.0000
Gait Speed (meters/second), mean	1.05 ± 0.01	1.02 ± 0.01	0.0891
METs/week of leisure-time physical activity			
0	38.5 ± 3.4	46.0 ± 3.3	0.0210
>0-<22.5	29.8 ± 2.3	27.9 ± 2.2	0.5112
22.5-<35	12.5 ± 1.2	12.0 ± 1.6	0.7407
≥35	19.1 ± 2.3	14.1 ± 1.6	0.0690
Smoking status			
Never	26.4 ± 2.0	56.0 ± 1.8	0.0000
Current	23.6 ± 2.1	15.3 ± 1.8	0.0001
Former	50.1 ± 2.3	28.7 ± 1.7	0.0000
Alcohol consumption			
Non-drinker	25.0 ± 2.3	40.1 ± 2.2	0.0000
Moderate drinker	59.3 ± 2.9	52.8 ± 2.3	0.0857
Heavy drinker	15.6 ± 1.9	7.1 ± 1.1	0.0010
Arthritis	36.0 ± 1.5	51.9 ± 2.1	0.0000
Walking aid	1.3 ± 0.3	2.1 ± 0.5	0.2207
BMI >30 kg/m^2^	29.7 ± 2.1	32.0 ± 2.4	0.3896
BMI (kg/m^2^), mean	28.2 ± 0.2	28.3 ± 0.3	0.8759
Waist circumference (cm), mean	103.4 ± 0.5	95.3 ± 0.8	0.0000
Systolic blood pressure (mm Hg), mean	129.5 ± 0.8	135.4 ± 0.6	0.0000
Diastolic blood pressure (mm Hg), mean	74.0 ± 0.5	71.3 ± 0.7	0.0025
Fasting Glucose (mg/dl), mean	112.7 ± 1.7	105.0 ± 1.4	0.0004
Triglyceride (mg/dl), mean	175.9 ± 11.3	158.9 ± 4.7	0.1573
Total cholesterol concentration (mg/dl), mean	207.5 ± 2.1	220.3 ± 1.5	0.0000
LDL cholesterol (mg/dl), mean	128.3 ± 1.7	130.3 ± 1.5	0.3398
HDL cholesterol (mg/dl), mean	46.7 ± 0.5	59.1 ± 0.8	0.0000

NHANES, National Health and Nutrition Examination Survey; SE, standard error; METs, metabolic equivalents; LDL, low-density lipoprotein cholesterol; HDL, high-density lipoprotein cholesterol.

**Table 2:** Prevalence of the individual components of the metabolic syndrome and the syndrome itself by gender and gait speed impairment status, NHANES 1999–2002

	Men				Women			
	
	Gait speed impairment				Gait speed impairment			
	
Metabolic disorder	Yes (N = 332) % ± SE	No (N = 503) % ± SE	P value	Age-adjusted P value^a^	Yes (N = 144) % ± SE	No (N = 706) % ± SE	P Value	Age-Adjusted P value^a^
Abdominal obesity	48.0 ± 3.4	53.4 ± 3.2	0.3128	0.5148	64.7 ± 5.2	67.8 ± 2.4	0.5827	0.2839
Hypertriglyceridemia	37.3 ± 3.5	42.8 ± 3.2	0.2035	0.6853	44.8 ± 5.1	42.5 ± 1.8	0.6813	0.3650
Low HDL cholesterol	31.7 ± 2.9	31.9 ± 2.2	0.9603	0.8271	44.2 ± 4.8	32.4 ± 2.3	0.0609	0.0001
High blood pressure or medication use	71.3 ± 3.8	59.6 ± 2.5	0.0182	0.2101	84.9 ± 4.3	68.6 ± 2.4	0.0035	0.1140
High fasting glucose or medication use	37.9 ± 2.4	31.7 ± 2.5	0.1333	0.3211	38.3 ± 4.6	20.4 ± 1.8	0.0005	0.0006
Metabolic syndrome	42.6 ± 2.7	39.2 ± 2.8	0.4330	0.4581	53.0 ± 5.2	44.5 ± 2.0	0.1166	0.0039

NHANES, National Health and Nutrition Examination Survey; SE, standard error; HDL, high-density lipoprotein.

^a^Age-adjusted to the 2000 U.S. standard population.

**Table 3:** Odds ratios of gait speed impairment due to the presence versus the absence of the individual components of the metabolic syndrome and the syndrome itself by gender, NHANES 1999–2002

Metabolic disorder	Unadjusted OR (95% CI)	Adjusted model 1^a ^ OR (95% CI)	Adjusted model 2^b ^ OR (95% CI)	Adjusted model 3^c ^ OR (95% CI)
Men				
Abdominal obesity	0.81 (0.53–1.24)	0.85 (0.52–1.37)	0.80 (0.49–1.32)	1.07 (0.65–1.76)
Hypertriglyceridemia	0.79 (0.55–1.14)	0.86 (0.58–1.30)	0.85 (0.53–1.36)	0.92 (0.54–1.56)
Low HDL cholesterol	0.99 (0.69–1.43)	0.98 (0.67–1.45)	1.05 (0.65–1.72)	0.83 (0.54–1.28)
High blood pressure or medication use	1.69 (1.08–2.64)	1.24 (0.76–2.03)	1.28 (0.77–2.13)	1.11 (0.65–1.89)
High fasting glucose or medication use	1.31 (0.91–1.89)	1.13 (0.75–1.70)	1.19 (0.79–1.79)	1.14 (0.73–1.76)
Metabolic syndrome	1.15 (0.80–1.65)	1.10 (0.73–1.66)	N/A	1.15 (0.76–1.74)^d^
Women				
Abdominal obesity	0.87 (0.53–1.44)	0.72 (0.42–1.21)	0.48 (0.26–0.89)	0.37 (0.18–0.76)
Hypertriglyceridemia	1.09 (0.70–1.71)	0.96 (0.57–1.59)	0.68 (0.36–1.30)	0.59 (0.31–1.10)
Low HDL cholesterol	1.65 (0.98–2.77)	1.88 (1.04–3.41)	2.26 (1.08–4.75)	2.45 (1.07–5.63)
High blood pressure or medication use	2.57 (1.22–5.39)	1.30 (0.61–2.77)	1.33 (0.61–2.92)	1.00 (0.45–2.22)
High fasting glucose or medication use	2.42 (1.60–3.68)	1.90 (1.14–3.17)	2.05 (1.12–3.74)	1.84 (0.84–4.03)
Metabolic syndrome	1.41 (0.91–2.17)	1.20 (0.75–1.91)	N/A	0.89 (0.51–1.53)^d^

NHANES, National Health and Nutrition Examination Survey; OR, odds ratio; CI, confidence interval; HDL, high-density lipoprotein; N/A, not applicable.

^a^Adjusted for age (continuous) and METs/week (0, >0-<22.5, 22.5-<35, ≥35).

^b^Additional adjustment for other components of the metabolic syndrome (each component: yes/no).

^c^Adjusted for all the above plus race/ethnicity (non-Hispanic white; non-Hispanic black; Mexican American; other), education (<high school, ≥high school), smoking status (current, former, never), alcohol consumption (non-drinker, moderate, heavy), arthritis status (dichotomous), and use of an assistive device (dichotomous).

^d^Adjusted for all of the above except the individual components of the metabolic syndrome (i.e., age [continuous], METs/week, race/ethnicity, education, smoking status, alcohol consumption, arthritis status, and use of an assistive device).
