# Supplementary material for: Association between the metabolic syndrome and its components and gait speed among U.S. adults aged 50 years and older: a cross-sectional analysis
Source: BMC Public Health. 2006 Nov 14;6:282. doi: 10.1186/1471-2458-6-282 (PMC1654157; doi:10.1186/1471-2458-6-282)
Supplement: Additional File 1 — Sex and age distribution of study participants and excluded participants, NHANES 1999–2002. Sex and age characteristics of participants included and excluded from the study. [file 1471-2458-6-282-S1.doc]

Additional file 1. Sex and age distribution of study participants and excluded participants, NHANES 1999-2002

|  | **Study participants**  **(N = 1685)**  **%** | **Excluded participants**  **(N = 230)**  **%** |
| --- | --- | --- |
| **Sex** |  |  |
| Male | 89.4 | 10.6 |
| Female | 86.7 | 13.4 |
| **Age, y** |  |  |
| 50-64 | 89.0 | 11.0 |
| ≥65 | 87.1 | 12.9 |

NHANES, National Health and Nutrition Examination Survey.
